# Recent Advances in Radical Prostatectomy: A Narrative Review of Surgical Innovations and Outcomes

**DOI:** 10.3390/cancers17050902

**Published:** 2025-03-06

**Authors:** Seon Beom Jo, Jong Wook Kim

**Affiliations:** 1 Department of Pharmacology, Korea University College of Medicine, Korea University, 73 Goryeodae-ro, Seongbuk-gu, Seoul 02841, Republic of Korea; rhfughfkddl@naver.com; 2 Department of Urology, Korea University Guro Hospital, Korea University College of Medicine, Seoul 08308, Republic of Korea

**Keywords:** prostate cancer, robotic-assisted surgery, radical prostatectomy, single-port robotics, Retzius-sparing technique, oncological outcomes, functional recovery, surgical innovation, urinary continence, quality of life

## Abstract

Prostate cancer is one of the most commonly diagnosed malignancies worldwide and is a major cause of cancer-associated morbidity in men. Radical prostatectomy (RP) is a cornerstone of intervention for organ-confined diseases and offers a potentially curative outcome. In recent decades, RP has undergone transformative changes, moving from open surgery, with significant morbidity, to minimally invasive and robot-assisted techniques. These advances have improved surgical precision, reduced blood loss, and accelerated functional recovery. Key enhancements, such as the “Veil of Aphrodite”, hood-sparing, and Retzius-sparing approaches, aim to preserve neurovascular structures vital for continence and sexual function, addressing the persistent challenge of balancing oncological control with quality-of-life outcomes. Single-port (SP) robotic platforms represent the latest frontier, offering various access routes, including extraperitoneal, transvesical, transperitoneal, and transperineal routes, to further reduce incisional morbidity. Early experiences with SP robot-assisted radical prostatectomy(RARP) suggest favorable continence rates and short hospital stays, although concerns remain regarding the technical complexity and potential margin status of the advanced disease. Comparisons across these techniques revealed broadly similar long-term oncological outcomes, underscoring the importance of patient selection, tumor staging, and surgeon expertise. Ongoing innovations in robotic systems, augmented imaging modalities, and personalized surgical planning are likely to refine prostate cancer care and enhance both survival and post-treatment quality of life.

## Simple Summary

Prostate cancer is a leading health concern among men worldwide. Traditional surgical treatments often result in significant side effects that affect the quality of life. Our research examines recent advancements in surgical techniques, such as robot-assisted procedures, aimed at effectively treating prostate cancer while minimizing adverse effects. By evaluating these innovations, we aimed to provide insights that can guide future research and improve patient outcomes.

## 1. Introduction

Prostate cancer is one of the most frequently diagnosed malignancies in men worldwide, with an increasing incidence as the population ages [1,2,3]. Radical prostatectomy (RP) has long been the cornerstone therapy for patients with organ-confined diseases, providing curative potential. Over the past few decades, RP has evolved from open approaches with significant morbidity to minimally invasive options that reduce complications and aim to preserve quality of life. Laparoscopic radical prostatectomy introduced smaller incisions and faster convalescence but was technically challenging due to limited instrument articulation and two-dimensional vision [4,5]. The advent of robot-assisted radical prostatectomy (RARP) at the turn of the century has transformed prostate surgery by enabling enhanced 3D visualization, wristed instrumentation, and stable camera control [6,7]. Currently, RARP has become the dominant modality in many centers across high-income nations and has led to reduced blood loss, shorter hospital stays, and early functional recovery than open surgery [8,9]. Despite these advancements, the pursuit of improving key outcomes such as urinary continence and sexual function continues. Surgeons have developed novel dissection planes and techniques (e.g., Retzius-sparing and hood-sparing) to preserve the periurethral or anterior supporting structures that are believed to be critical for early continence recovery [10,11,12,13]. Most recently, single-port (SP) robotic platforms have been adopted, expanding the potential approaches (extraperitoneal, transperitoneal, perineal, and transvesical) to reduce incisional morbidity [14,15,16]. The continued evolution of surgical techniques and technologies in radical prostatectomy reflects the ongoing efforts to balance oncological control with the preservation of quality of life. This review examines the latest advances in radical prostatectomy, emphasizing innovations in techniques and outcomes that have shaped current clinical practice. By understanding the progression from open to robot-assisted and single-port approaches, as well as comparing emerging techniques such as hood-sparing and Retzius-sparing, this narrative review aims to provide a comprehensive overview for clinicians seeking to optimize prostate cancer management.

## 2. The History and Evolution of Radical Prostatectomy

The treatment of prostate cancer dates back to 1904 when Hugh Hampton Young in the United States performed the first radical prostatectomy using the perineal approach. Despite its significant limitations, including high blood loss, this procedure is recognized as the foundation for modern prostate cancer surgery [17]. In 1945, British surgeon Terence Millin introduced the retropubic approach, which improved surgical visualization and reduced bleeding, enhancing the safety of the operation [18]. However, it still carries significant side effects, such as impotence and urinary incontinence. In 1982, Dr. Patrick Walsh developed nerve-sparing radical prostatectomy, which significantly reduced postoperative erectile dysfunction and urinary incontinence [19]. In 1992, Schuessler et al. performed the first laparoscopic radical prostatectomy (LRP), which offered advantages such as reduced blood loss and faster recovery [20]. However, the learning curve was prolonged, and Neurovascular Bundle (NVB) preservation proved challenging because of the limitations of 2D visualization and restricted instrument maneuverability [21]. In 2000, Dr. Mani Menon performed the first da Vinci robot-assisted radical prostatectomy (RARP), ushering in a new era of precision in prostate cancer surgery that has shown clinical effectiveness in reducing complications and improving outcomes [22].

The anterior approach (access to the Retzius space) has recently become the standard of care approach for RARP. However, modified techniques, such as the posterior approach (Retzius-sparing) and the Hood technique, aimed at maximizing the recovery of urinary continence and sexual function, are gaining attention [10,12,23]. Single-port robotic systems are the latest innovations seeking to minimize the number of ports or incisions used while retaining the established benefits of RARP [14,15,16].

## 3. A Spectrum of Surgical Approaches in Robot-Assisted Radical Prostatectomy: Standard, Hood, Lateral, and Retzius-Sparing Techniques

### 3.1. Standard (Anterior) RARP and Its Refinements

**Figure 1:**
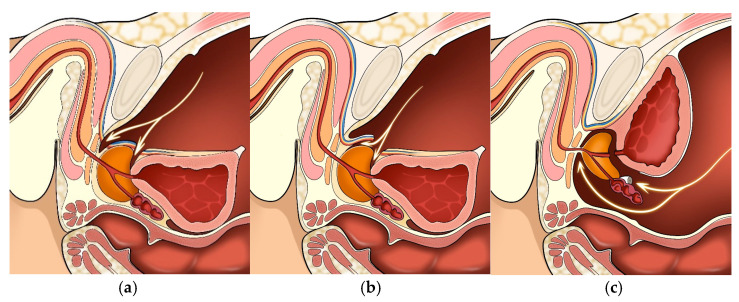
(**a**) Conventional (anterior) approach: illustration showing a standard anterior route that begins with incising the endopelvic fascia and entering the retropubic (Retzius) space. This straightforward orientation directly visualizes the bladder neck, apex, and associated neurovascular structures. (**b**) Hood technique (anterior) depiction of an anterior approach that extends the dissection plane more proximally, preserving a broader fascial cuff around the external sphincter. This “hood” of tissue aims to optimize periurethral support, potentially enhancing early continence and maintaining oncologic safety by sparing key neurovascular fibers. (**c**) Retzius-sparing (posterior) approach: diagram illustrating a posterior route where the retropubic (Retzius) space remains intact, leaving the dorsal venous plexus, puboprostatic ligaments, and endopelvic fascia undisturbed. Dissection continues behind the bladder in a reversed orientation toward the prostate apex by preserving critical anterior support structures.

When performing nerve-sparing robot-assisted radical prostatectomy (RARP) via the anterior route, surgeons typically incise the endopelvic fascia and enter the Retzius space to gain excellent visualization of critical landmarks, including the bladder neck and dorsal venous plexus (Figure 1a). By controlling the dorsal venous complex (DVC) and working sequentially toward the apex, the operator can clip the pedicles precisely and methodically dissect the prostate by controlling the dorsal venous complex and working sequentially toward the apex. Recognizing that neurovascular bundles (NVBs) extend more anteriorly than once presumed, potentially up to the 2 and 10 o’clock positions, Menon and Kaul pioneered the so-called “Veil of Aphrodite” in the mid-2000s [13,24]. Their technique involved meticulous release of the anterolateral prostatic fascia and gentle “peeling” of the NVB off the prostate to preserve a generous veil of tissue, thereby decreasing traction or thermal injury to key nerves [9,24]. Subsequent experience indicated that even greater periurethral coverage might further improve urinary control, prompting additional refinements.

A major series of standard RARP has been documented to have consistent and favorable results. Positive surgical margin (PSM) rates in organ-confined disease (pT2) typically range from ~10–20%, while ~40–60% of patients achieve zero-pad continence at 1–3 months, increasing to ≥85% by 1 year [9,25,26]. Nerve-sparing is performed at the lateral aspects of the prostate to preserve erectile function, with approximately 50–70% of men recovering adequate erections if the preoperative function is normal [27]. Surgeons adopt either an interfascial plane (partial nerve-sparing) or an intrafascial plane (aggressive nerve-sparing) based on preoperative risk and MRI findings [5].

### 3.2. Hood Techniques

Building on the basic anterior approach, Wagaskar et al. recently described the “Hood” technique [10]. This approach extends the high anterior release such that a broader cuff of fascia is preserved near the external sphincter, effectively creating a “hood-like” tissue shield at the prostate apex (Figure 1b). By shifting the anterior boundary of the dissection more proximally, the Hood technique guards the additional anterior and periurethral neurovascular fibers while maintaining oncological safety. The impetus is to maintain a robust hammock of the membranous urethra, potentially improving early continence. In a large single-center series, hood-sparing RARP achieved ~80% dryness at 1 month and ~90% dryness at 3 months with minimal impact on margin rates [10]. Some believe that the hood-sparing approach further complements meticulous nerve sparing by ensuring that the apical dissection remains close to the prostate capsule while leaving intact periurethral support. As a result, “hood technique” data show 6–16% PSM rates in pT2 disease [10,11,28]. The transition from standard to hood-sparing RARP is anatomically straightforward for surgeons who are already comfortable with the anterior approach. While Retzius sparing (described below) requires a posterior route, the hood technique simply modifies the standard anterior route by limiting the wide mobilization of retropubic structures.

### 3.3. Lateral Approach

When performing nerve-sparing robot-assisted radical prostatectomy (RARP) via the lateral route, surgeons typically proceed medially toward the prostate base and apex [29,30,31]. Early anatomical studies revealed that approximately 20–25% of periprostatic nerves lie closer to the ventral and lateral aspects of the prostatic capsule, highlighting the potential advantage of a lateral dissection to optimize nerve preservation [32].

From a technical standpoint, the lateral approach can be more challenging for surgeons primarily trained in anterior or posterior approaches. However, it can preserve the anterior pubovesical complex (dorsal vein complex, puboprostatic ligaments) by avoiding wide mobilization of the retropubic space, potentially accelerating early continence. Rodríguez Socarrás et al. found that ~85–86% of patients in a large (*n* = 513) cohort achieved immediate continence, with PSM rates of 33–38% in organ-confined disease [29]. A single-surgeon experience in 70 patients by Giulioni et al. reported a 15% PSM rate, 81% continence at 6 weeks (rising to 94% by 12 months), and an 84% potency rate at 1 year [30]. Nonetheless, as these authors note, a steep learning curve remains a concern, especially in higher-BMI patients or those with large prostates.

Overall, the lateral approach appears to balance effective oncological control with robust nerve-sparing for urinary and sexual function. Additional randomized studies are needed to clarify its long-term benefits relative to standard or Retzius-sparing approaches, but current evidence suggests it may serve as an alternative or complementary method in specialized centers [30,31].

### 3.4. Retzius-Sparing (Posterior) Access

In 2010, Bocciardi et al. (and subsequently Galfano et al.) popularized the concept of a Retzius-sparing approach to maintain the entire anterior compartment (puboprostatic ligaments, dorsal vein complex, and endopelvic fascia) intact [12,23]. This approach involved incising the peritoneum posteriorly near the cul-de-sac behind the bladder, exposing the seminal vesicles first, and then working antegrade along the prostatic capsule. The apex was dissected in a reversed orientation, culminating in a vesicourethral anastomosis without disturbing the retropubic space (Figure 1c) [12,23,33].

The reported functional benefit is an earlier return to continence because normal anatomic support for the sphincter is not disrupted [23,34]. Several prospective or randomized series have confirmed approximately 65–80% immediate or 1-week dryness, surpassing typical anterior results [33,34,35]. These differences narrow at 12 months, with many studies reporting that standard and Retzius-sparing outcomes converge. There are contradictory reports suggesting the possibly higher PSM rates in advanced or anteriorly located tumors, presumably because apical dissection under a reversed angle can be challenging [12,33,34]. Nonetheless, a large series from experienced centers showed that Retzius-sparing can be safely performed for low-to intermediate-risk disease with no deterioration in mid-term oncological endpoints [33,36].

This posterior approach is conceptually more demanding for surgeons trained in anterior RARP and can be less intuitive in handling large median lobes or advanced T3 lesions. Some surgeons prefer Retzius-sparing, primarily in cases where early continence is paramount, and tumor extension is not suspected near the apex or anterior region [12,33,34].

## 4. Optimizing Functional Recovery: Bladder Neck, Urethral, and ARVUS Techniques

While significant progress has been made with refined surgical techniques, post-prostatectomy incontinence remains a persistent challenge. In response, further innovations have focused on preserving the bladder neck, maximizing functional urethral length, and employing advanced reconstructions such as the Advanced Reconstruction of Vesicourethral Support (ARVUS) method. These strategies are designed to expedite early urinary continence by safeguarding or restoring the critical pelvic support structures that underlie sphincter function.

### 4.1. Bladder Neck Preservation (BNP)

Bladder neck preservation involves careful dissection around the prostate base, sparing as much of the bladder neck musculature as possible. By maintaining the native circular fibers of the bladder neck, surgeons aim to preserve an essential component of urinary continence. Meta-analyses of both open and minimally invasive series suggest that BNP significantly reduces time to continence, with improvements in 1-week and 1-month pad-free rates [24,25,34,35]. This approach can be anatomically challenging, particularly in patients with larger glands or complex apical disease, yet when feasible, it may hasten continence recovery without negatively impacting cancer control. Surgeons must balance the risk of leaving residual prostatic tissue if the dissection is overly conservative, emphasizing the importance of preoperative imaging and careful patient selection [24,25].

### 4.2. Urethral Preservation

In parallel with BNP, several centers emphasize maximal urethral preservation—particularly near the prostatic apex—to preserve the intrinsic sphincter and length of the membranous urethra [10,23,36]. Prostate apex dissection carried out too far distally may shorten the urethra or disrupt the rhabdosphincter complex, delaying continence. Conversely, a carefully executed apex release (e.g., “peeling off” the apex at the correct plane, with the dorsal venous complex individually suture-ligated or selectively controlled) can maximize functional urethral length. Preliminary data suggest that such approaches yield a higher proportion of patients with zero-pad or one-pad usage in 1–3 months [27,36,37]. The exact advantage of urethral preservation may depend on the tumor stage, preoperative function, and the surgeon’s ability to maintain adequate margins at the apex.

### 4.3. The ARVUS Technique

Alongside direct bladder neck and urethral preservation, certain authors advocate specialized reconstructions (e.g., the Advanced Reconstruction of Vesicourethral Support [ARVUS]) to bolster peri-urethral support. ARVUS was originally described by Student et al. as a “semi-circular” or “hammock” reconstruction, in which levator ani fibers, Denonvilliers fascia, and the median dorsal raphe are sutured around the urethrovesical anastomosis [38,39]. This maneuver effectively recreates a dynamic support akin to a sling, restoring normal anatomic relationships that may be lost after prostate removal. Prospective trials and early cohorts have reported improved early continence (up to 40–60% pad-free at 2–4 weeks) and no detrimental effect on surgical margin rates [38,39,40]. Additional validations by Kováčik et al. further confirm that ARVUS does not significantly lengthen operative times while offering robust support for the vesicourethral junction [39].

## 5. Single-Port RARP and Novel Access Routes

### 5.1. Rationale and Initial Adoption

Single-port (SP) robotics were introduced to reduce incisional morbidity further and theoretically improve pain control and cosmesis by consolidating all instruments into a single 2–4 cm incision. Single-site laparoscopic radical prostatectomy has been attempted with standard multiarm robots via a single gel port; instrument crossing and suboptimal triangulation are major pitfalls [14,41,42]. The da Vinci SP system (Intuitive Surgical, Sunnyvale, CA, USA) overcomes many of these issues by providing multiple double-jointed instruments that pass coaxially, thereby permitting intra-abdominal articulation [15,16]. SP-RARP can replicate standard- or Retzius-sparing planes. Surgeons can also place the port extraperitoneally, transperitoneally, perineally, or transvesically, each bearing specific advantages and challenges [15,43].

### 5.2. Extraperitoneal and Transperitoneal Approach

The extraperitoneal approach with the SP helps avoid bowel manipulation and may facilitate a shorter hospital stay. Early cohort studies from Kim et al. and Kaouk et al. reported minimal use of additional ports, short operative times, and safe PSM rates of approximately 20–30% for pT2 disease, with 60–80% dryness at 1–3 months [43,44,45]. Meanwhile, the transperitoneal SP method more closely parallels the standard multiport RARP, albeit channeled through a single umbilical incision. Studies have shown no substantial differences in long-term oncological or functional outcomes relative to multiport RARP, although the learning curve can be steeper if surgeons rely solely on a single cannula for retraction [15,43].

### 5.3. Transperineal Single-Port

The perineal route with a single-port cannula attempts to replicate the historical perineal open prostatectomy but with robotic articulation. It helps avoid abdominal incisions, which can be beneficial for patients undergoing multiple laparotomies or ostomies. However, a tight operative field may hamper wide or extended pelvic lymph node dissection (ePLND). A small case series from Lenfant et al. and Yu et al. suggested that the approach is feasible but can yield higher PSM rates if not carefully performed [46,47]. Currently, single transperineal port placement remains a niche approach.

### 5.4. Transvesical Single-Port

Among SP variations, transvesical RARP, popularized by Kaouk et al., Zhou et al., Deng et al., and others, stands out because of its Retzius-sparing properties. Instead of incising the retropubic space, the surgeon places a ~3 cm suprapubic incision, incises the bladder dome by ~2 cm, and “floating-docks” the SP cannula inside the bladder [16,48,49,50]. The vantage point is intravesical; the bladder neck is incised circumferentially, the seminal vesicles reach posteriorly (akin to a posterior approach), and the apex is dissected from the inside of the bladder. The anterior support structures remained undisturbed without opening the retropubic space [16,48].

Some small-to-moderate series showed extremely promising early continence, immediate dryness, or near dryness in up to 80–90% of patients after Foley catheter removal [48,50,51,52]. For instance, Kaouk et al. initially reported a small series of 10 patients who underwent transvesical single-port RARP, most of whom were discharged within 24 h, with minimal pain medication and approximately 50% immediate continence. Deng et al. studied 60 patients with 15% PSM and 90% dryness at removal and noted no major complications or 30-day readmissions [50]. Zhou et al. described 35 patients with 91% immediate continence and a 4/35 PSM rate (11.4%); none of them had biochemical recurrence at early follow-up [51]. In a more recent multi-institutional collaboration, the same group reported consistent findings, although PSM in higher-stage diseases increased. Similarly, Chung et al. documented continence in four patients(100%), although two (50%) had advanced pT3 disease with positive margins [52].

Although the technique yields excellent early functional outcomes, it may be suboptimal for advanced or large-lobe disease owing to the limited working space inside the bladder and the reversed orientation for apex control. Surgeons must also adopt a new technique for pelvic lymph node dissection, as full ePLND is challenging transvesically. Nonetheless, transvesical single-port RARP may be ideal for smaller prostates in patients with prior abdominal surgeries or for those prioritizing a rapid return of continence [48,50,51,52].

## 6. Clinical Outcomes Across Multiple Approaches: A Comparative Table

Table 1 provides an updated summary table incorporating data from various centers and the column details approach, study sample, approximate pT2 vs. pT3 distribution, positive margin rates, urinary continence (UC) in the short term (commonly 1–3 months), potency if reported, and short-term biochemical recurrence (BCR) references. Because definitions vary (for dryness, some groups use “0 pads”, others “0–1 pad”, etc.), comparisons are approximate.

## 7. Discussion

Recent advances in radical prostatectomy have led to the development of a diverse array of surgical techniques that aim to maximize oncologic control while improving postoperative quality of life by enhancing urinary continence and sexual function [44,56]. In our review, we describe a spectrum of approaches ranging from the standard anterior robot-assisted radical prostatectomy (RARP) to more complex modifications—including hood-sparing, lateral, Retzius-sparing, and emerging single-port (SP) methods—with each technique offering its own balance of technical demands and potential benefits.

A key consideration is the learning curve associated with each approach. Comparison data indicate that the standard anterior RARP, which uses well-defined anatomical landmarks and allows a straightforward dissection in the Retzius space, has a more gradual learning curve compared to advanced modifications such as lateral or Retzius-sparing techniques [57,58,59,60]. The standard approach is thus particularly suitable for residents and novice surgeons, as it provides a solid foundation of robotic skills and a comprehensive understanding of pelvic anatomy before progressing to more technically challenging methods. In contrast, techniques like Retzius-sparing and hood-sparing RARP, although they may offer improvements in early continence and nerve preservation, demand a steeper learning curve due to their reversed dissection planes and more confined operative fields [58,59]. Consequently, when designing training programs, it is advisable to begin with the standard anterior approach and introduce more advanced techniques only after a baseline proficiency has been achieved.

Operator-dependent factors significantly affect postoperative outcomes. Technical proficiency in nerve-sparing dissection, precise preservation of periurethral support structures, and high cumulative case volume are essential for optimizing urinary continence and erectile function [61,62]. Likewise, patient-dependent factors—including age, body mass index (BMI), prostate size, and tumor stage—are crucial determinants of recovery. For example, patients with obesity or larger prostates may benefit from surgical approaches that reduce tissue trauma and operative time, while those with anterior tumors or advanced disease require meticulous preoperative planning to balance oncologic clearance with functional preservation [60,61].

While robotic technology has dramatically transformed surgical practice over the past 25 years, long-term studies have yet to conclusively demonstrate that RARP offers superior oncologic or functional outcomes compared to open radical prostatectomy [63]. Moreover, emerging alternative treatments—such as stereotactic radiotherapy and focal therapy—are gaining attention because of their potential to cure prostate cancer while minimizing complications like incontinence and erectile dysfunction; however, current evidence remains inconclusive and warrants further investigation [64,65,66]. This underscores the need for additional randomized studies, standardized training protocols, and technological advancements (including improved haptic feedback, augmented reality guidance, and patient-specific surgical planning) to fully realize the benefits of robotic surgery.

In summary, the selection of a surgical approach for radical prostatectomy should be tailored to individual patient characteristics (e.g., obesity, tumor location, and stage) as well as the surgeon’s experience. For novice surgeons, the standard anterior RARP remains the most accessible and recommended initial approach due to its forgiving learning curve and consistent outcomes [57,60]. As experience accumulates, advanced techniques such as Retzius-sparing and hood-sparing approaches can be gradually incorporated to further enhance early functional recovery. Concurrently, individualized reconstruction strategies—including bladder neck and urethral preservation with methods such as ARVUS—are essential for optimizing outcomes in patients with complex pelvic anatomies [60,61]. Ultimately, although robotic surgery has advanced considerably, its long-term advantages over open radical prostatectomy have yet to be definitively proven, emphasizing the need for continued comparative research, enhanced surgical training, and integration of emerging technologies.

## 8. Conclusions

The evolution of radical prostatectomy—from open surgery to diverse robot-assisted techniques—reflects a sustained effort to balance oncologic efficacy with quality-of-life preservation. While modified approaches such as hood-sparing, lateral, and Retzius-sparing RARP show promise in enhancing early functional recovery, they require higher technical expertise and have steeper learning curves. The standard anterior RARP remains the recommended initial approach for residents and novice surgeons, providing a solid foundation for the future adoption of advanced techniques. Future research should emphasize comparative outcomes, refinement of training protocols, and the incorporation of innovative technologies to further individualize treatment and improve both oncologic and functional outcomes.

## Figures and Tables

**Table 1:** Summary of clinical outcomes by RARP approaches.

Approach	Study (Year)	N	Stage pT2/pT3 (%)	PSM (%)	Early Continence	Potency (≥6 mo)	BCR or PSA	Notes
Standard (Anterior)	Ficarra et al. (2009) [5]	4000+	Mixed	10–20 (pT2)	~40–60% at 1–3 mo	~50–70% vary.	~5–10% at 1–2 y	Systematic review comparing laparoscopic/robotic vs. open.
	Menon et al. (2009) [53]	1100	~80/20	15–20	45–55% at 1 mo, ~90% at 1 y	~70–75% at 12 mo	~5–8%	“Veil of Aphrodite” pioneer.
	Patel et al. (2011) [54]	4000	~76.2/22.9	10.8% (overall)	67.7% at 6 wk, 85.4% at 3 mo	91.5% at 1 y	9.5% at 12 mo	Multi-institutional study; reports pentafecta outcomes.
Hood-Sparing	Wagaskar et al. (2021) [10]	300	~75/25	6	80% at 1 mo, 91% at 3 mo	~70–80% (1 y)	~5% short	Hood technique preserves periurethral structures.
	Shimmura et al. (2023) [11]	42	~70/30	16	69% at 1 mo, 91% at 3 mo	Not precisely stated	Not reported	Umbilical lig. + Hood; small single-center.
Retzius-Sparing (Posterior)	Galfano et al. (2013) [23]	200	~80/20	15–20	70–80% immediate to 1 mo	~65–75% at 1 y	~5–6%	Pioneer posterior approach.
	Dalela et al. (2017) [33]	100	85/15	18	71% vs. 47% (std) at 1 mo	~70% at 1 y	5–6% ~1 y	Pragmatic RCT favoring RS for early continence.
	Egan et al. (2021) [35]	70	80/20	34.3	95% dryness at 1 y	65.7% at 1 y	~4–5%	Possibly advanced disease.
Lateral	Rodríguez Socarrás et al. (2023) [29]	513	~70/30	33–38	~85–86% immediate dryness	Not reported	Not reported	Large cohort; good short-term UC; steep learning curve.
	Giulioni et al. (2024) [30]	70	~60/40	15	81% dryness at 6 wk, 94% at 1 y	84% at 1 y	Not reported	Single-surgeon experience; stable PSM .
Single-Port (SP)								
(Transvesical)	Deng et al. (2021) [50]	60	90/10	15	90% dryness at removal	60–70% at 3–6 mo	~5% BCR at 12 month	Transvesical retzius-sparing advantage.
	Zhou et al. (2020) [51]	35	80/20	11.4	91% immediate in smaller series	~65–70% 3–6 mo	No BCR short	No recurrences short term.
	Chung et al. (2024) [52]	4	50/50	50	100% continence at removal, safety pad at 1 mo	Not measured fully	1 persistent PSA	Very small case series; 2 advanced diseases.
	Ramos-Carpinteyro (2023) [48]	100	~80/20	~20?	80–90% immediate dryness	~65–70% (≥6 mo)	Not fully stated	First 100 transvesical SP cases from a single center.
(Extraperitoneal)	Abou Zeinab et al. (2023) [55]	238	~75/25	~29 (EP)	70% dryness at 1 mo (extraperitoneal)	~60–65% potency	~9% BCR short	Large multi-institution EP vs. TP matched.
	Kim et al. (2022) [44]	157	80/20	29	70% immediate dryness	64.4% at 9 mo	8.3% BCR at 9 mo	Extraperitoneal SP approach.
(Transperineal)	Lenfant et al. (2021) [47]	26	~70/30	65.4	75% dryness at 3 mo	Not stated	~4% BCR	Single-port perineal route.
	Yu et al. (2023) [46]	50	~85/15	10	48–80% dryness by 3 mo, 96–100% at 6–12 mo	Not stated	1 BCR at 12 month	Another single-surgeon series.

Abbreviations: PSM, positive surgical margin; UC, urinary continence; BCR, biochemical recurrence; EP, extraperitoneal; TP, transperitoneal.

## Abbreviations

The following abbreviations are used in this manuscript:

DVC	Dorsal venous complex
LRP	Laparoscopic radical prostatectomy
PSM	Positive surgical margin
RARP	Robot-assisted radical prostatectomy
RP	Radical prostatectomy
SP	Single-port
UC	Urinary continence
